# Application of a hybrid algorithm of LSTM and Transformer based on random search optimization for improving rainfall-runoff simulation

**DOI:** 10.1038/s41598-024-62127-7

**Published:** 2024-05-16

**Authors:** Wenzhong Li, Chengshuai Liu, Caihong Hu, Chaojie Niu, Runxi Li, Ming Li, Yingying Xu, Lu Tian

**Affiliations:** School of Water Conservancy and Transportation, Zhengzhou University, Zhengzhou, 450001 China

**Keywords:** Deep learning, Flood forecasting, Rainfall-runoff, RS-LSTM-transformer, Random search optimization, The middle reaches of the Yellow River, Hydrology, Natural hazards, Computer science

## Abstract

Flood forecasting using traditional physical hydrology models requires consideration of multiple complex physical processes including the spatio-temporal distribution of rainfall, the spatial heterogeneity of watershed sub-surface characteristics, and runoff generation and routing behaviours. Data-driven models offer novel solutions to these challenges, though they are hindered by difficulties in hyperparameter selection and a decline in prediction stability as the lead time extends. This study introduces a hybrid model, the RS-LSTM-Transformer, which combines Random Search (RS), Long Short-Term Memory networks (LSTM), and the Transformer architecture. Applied to the typical Jingle watershed in the middle reaches of the Yellow River, this model utilises rainfall and runoff data from basin sites to simulate flood processes, and its outcomes are compared against those from RS-LSTM, RS-Transformer, RS-BP, and RS-MLP models. It was evaluated against RS-LSTM, RS-Transformer, RS-BP, and RS-MLP models using the Nash–Sutcliffe Efficiency Coefficient (NSE), Root Mean Square Error (RMSE), Mean Absolute Error (MAE), and Bias percentage as metrics. At a 1-h lead time during calibration and validation, the RS-LSTM-Transformer model achieved NSE, RMSE, MAE, and Bias values of 0.970, 14.001m^3^/s, 5.304m^3^/s, 0.501% and 0.953, 14.124m^3^/s, 6.365m^3^/s, 0.523%, respectively. These results demonstrate the model's superior simulation capabilities and robustness, providing more accurate peak flow forecasts as the lead time increases. The study highlights the RS-LSTM-Transformer model's potential in flood forecasting and the advantages of integrating various data-driven approaches for innovative modelling.

## Introduction

In recent years, the intensification of global climate change and human activities has resulted in an increase in unpredictable extreme weather events such as floods, droughts, and storms^1,2^. Among these events, the impact of flood disasters has surpassed national boundaries and become a global concern^3–5^. The frequency, intensity, and scale of flood disasters continue to rise, posing significant risks and threats to human society, the economy, and the environment. Therefore, accurate flood prediction and timely implementation of corresponding protective measures have become crucial^6–8^. The simulation and analysis of rainfall-runoff processes play a vital role in flood forecasting and watershed water resources management^9^, especially for long-term predictions^10^. However, due to the presence of multiple complex physical processes, including spatiotemporal variations in rainfall-runoff transformation, spatial heterogeneity of watershed land surface characteristics, and routing behavior of runoff^11–13^, flood processes exhibit nonlinearity, non-stationarity, spatiotemporal variability, and complex mechanisms of runoff formation^14^. This complexity poses challenges in rainfall-runoff simulation, making it a challenging task.

In the past few decades, various methods and models have been developed to simulate rainfall-runoff processes in flood forecasting, including physical models, conceptual models, and data-driven models^15,16^. Physical and conceptual models, also referred to as process-based models, employ empirical and analytical equations derived from physical phenomena^17–19^. These traditional hydrological models simulate rainfall-runoff and other hydrological processes by incorporating physical mechanisms or concepts, offering valuable insights into understanding watershed runoff^20–22^. Nevertheless, their practical application is limited due to the extensive requirement of hydrological and land surface data, as well as the need for accurate understanding of the runoff generation process^23–25^.

In contrast, data-driven models have the ability to capture the relationship between meteorological data and runoff without relying on explicit knowledge of the physical behavior of hydrological systems^26^. These models are capable of extracting intrinsic connections from large datasets and learning the corresponding relationships among variables^27^. Consequently, they can simulate highly nonlinear and non-stationary relationships in hydrological systems^28^.

With the advancement of technology, data-driven models have gained increasing attention in the era of artificial intelligence and big data, and they have been widely applied in hydrology for runoff simulation^29^. Specifically, artificial neural networks (ANN) have shown high accuracy in modeling complex rainfall-runoff processes^30,31^, and perform comparably to physical models^32^. Research has also found that compared to commonly used regression models, ANN can provide more accurate predictions of runoff^33^. Yan et al. have scientifically predicted mid to long-term runoff by integrating a combination of different climatic factors into an improved BP model^34^*.* Though, ANN also has limitations, such as a lack of memory^20,35^, as it lacks an internal mechanism to handle sequential data, such as floods. This means that ANN cannot effectively capture temporal dependencies. In addition to ANN, other methods such as support vector machines (SVM), adaptive neuro-fuzzy inference systems (ANFIS), and multilayer perceptron (MLP) can be used to address modeling and optimization problems in flood forecasting^36–38^. Gao et al. have utilized a hybrid model combining the Soil and Water Assessment Tool (SWAT) with a Multilayer Perceptron (MLP) for runoff prediction, demonstrating high efficiency^39^.

Recurrent neural networks (RNNs) have been demonstrated to accurately and effectively handle time series data, addressing the limitations of artificial neural networks (ANNs) in this aspect^40^, which renders them as an efficient approach for simulating intricate dynamic hydrological processes^15^. In the late 1990s, a more modern architecture of RNN called Long Short-Term Memory (LSTM) was proposed^41^. As a significant advancement in the field of deep learning, LSTM addresses the issue of vanishing or exploding gradients that traditional RNNs face when dealing with long sequences by introducing gate mechanisms and memory cells. It captures and remembers the temporal dynamics of model inputs and processes data in sequential order, allowing for better capturing of long-term dependencies^42^. Currently, LSTM has been widely applied in various fields such as natural language processing, stock market prediction, and speech recognition^43,44^.

In recent years, there has been significant progress in the application of LSTM-based methods in flood forecasting, making them important technologies for river, reservoir, and urban flood prediction^45–47^. Prior research has highlighted the distinctive capabilities of LSTM methods in simulating rainfall-runoff processes^48^. Analysis of historical flood data and meteorological factors allows LSTMs to accurately predict flood trends, significantly aiding flood prevention efforts^49,50^. Man et al. has developed an enhanced LSTM model, markedly improving the accuracy of peak daily runoff predictions^51^. Yao et al. have devised a dynamic, highly accurate composite runoff model combining Adaptive Weighting Module (AWM), Convolutional Neural Networks, Gated Recurrent Units, and LSTM^52^. Despite these advancements, LSTM models require high-quality, substantial data, and hyperparameter optimization remains crucial for optimal simulation results^53^.

The introduction of attention mechanisms has been a significant breakthrough in the field of neural networks, providing an effective solution to the selective focus problem in information processing^48^. By automatically learning the intrinsic correlations and importance of data, attention mechanisms allow models to selectively focus on relevant parts for the task at hand. This enables better capturing of the relationships and important information among inputs and facilitates more in-depth processing. The advent of the Transformer model in 2017 gained considerable recognition due to its support for parallel computation, fast training capabilities, and effective modeling of both short-term and long-term dependencies. It has shown promising results when applied to time series data analysis^54^. Nonetheless, to achieve better time series predictions for different tasks, researchers in the field have made various improvements to the Transformer model^55–57^. These improvements underscore the Transformer model's predictive prowess across diverse domains. Recent investigations have highlighted the superiority of the Transformer model over LSTM in long-term hydrological forecasting^58,59^. Yet due to the self-attention mechanism employed in both the encoder and decoder of the Transformer, it has high computational space complexity and weak perception of local information features. This makes the model susceptible to the influence of outliers, which needs further optimization considerations^60^. Despite this, the Transformer model exhibits great potential in the field of hydrological forecasting and warrants continued exploration and application, similar to LSTM.

In this study, we explore the application of the RS-LSTM-Transformer coupled model in the field of flood prediction models. It focuses on the Jingle River watershed in the middle Yellow River, China, as a representative case. We developed the RS-LSTM-Transformer hybrid flood forecasting model by modifying the internal structure of the Transformer model and integrating it with LSTM and the RS random search algorithm. This model was validated using 98 real flood events. Performance metrics such as Nash–Sutcliffe Efficiency (NSE), Root Mean Square Error (RMSE), Mean Absolute Error (MAE), and Bias were employed to evaluate the model's performance, with comparisons drawn against RS-LSTM, RS-Transformer, RS-BP, and RS-MLP models. The RS-LSTM-Transformer hybrid model aims to address significant challenges in simulating peak flow errors and enhancing the robustness of flood predictions, thus providing a scientific basis for flood control and mitigation in the watershed.

## Methods

### RS algorithm

Randomized Search (RS) algorithm was proposed in 2012 as a more efficient alternative to traditional Grid Search algorithm for exploring hyperparameter space. Unlike grid search, RS introduces randomness into the search process, enabling a more efficient exploration of the hyperparameter space^61^. In order to achieve optimal model performance, RS was utilized to optimize the parameters of the LSTM-Transformer model, as well as the parameters of the LSTM, Transformer, BP, and MLP models for comparison. The framework for parameter optimization using the RS algorithm mainly comprises three steps:

*Step 1*: Determine the search space for each model's parameters and set initial parameter values. Preliminary experiments were conducted through manual parameter tuning to identify key parameters that significantly impact the models, considering the large number of parameters and the need for efficiency and fairness. The preliminary results revealed that the number of attention heads is crucial for LSTM-Transformer and Transformer, while the number of neurons plays a key role in LSTM, BP, and MLP models. Moreover, common adjustable parameters including time steps, batch size, and the number of cells were selected for all five models.

*Step 2*: The RS algorithm was employed to search for and optimize the selected parameters for each of the five models. *NSE* was utilized as the performance evaluation metric to assess the models' performance and identify the optimal parameter combinations for each model.

*Step 3*: Construct the model with the best performance using the optimal parameter combinations obtained from Step 2.

**Figure 1:**
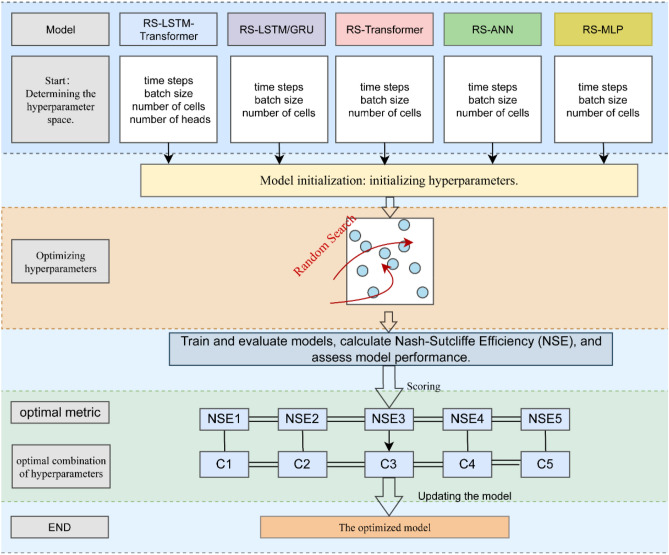
The RS optimization algorithm was employed to search for optimal parameter combinations in a comparative framework that included LSTM-Transformer, LSTM, Transformer, BP, and MLP models. Through experimental methods, the framework identified both key parameters and the best parameter combinations.

As shown in Fig. 1, the framework outlines the process of parameter optimization for the five models using the RS algorithm. C1 to C5 represent the optimal parameter combinations for different models.

### Improved transformer

In the field of flood forecasting, it has been observed that the original Transformer model has limitations when applied to multivariate time series data. The traditional Transformer model is based on an encoder-decoder architecture with attention mechanisms^54^. In translation tasks, positional encoding is utilized to capture the positional information of data points, which is crucial for accurate translation. Moreover, the decoder is responsible for decoding and generating information from the encoded data. However, for flood time series data, which involves predicting future values using multiple variables as inputs, the significance of positional encoding diminishes, and there is no requirement for parallel computation using a decoder structure. To better adapt the Transformer model to the task of flood forecasting, several improvements have been implemented. Firstly, positional encoding and the decoder part have been removed since positional encoding has minimal impact on flood time series data. Secondly, the internal structure of the model has been adjusted by incorporating convolutional layers and global average pooling structures to effectively capture local features in the time series. Lastly, a fully connected layer has been employed to generate the forecasting results.

**Figure 2:**
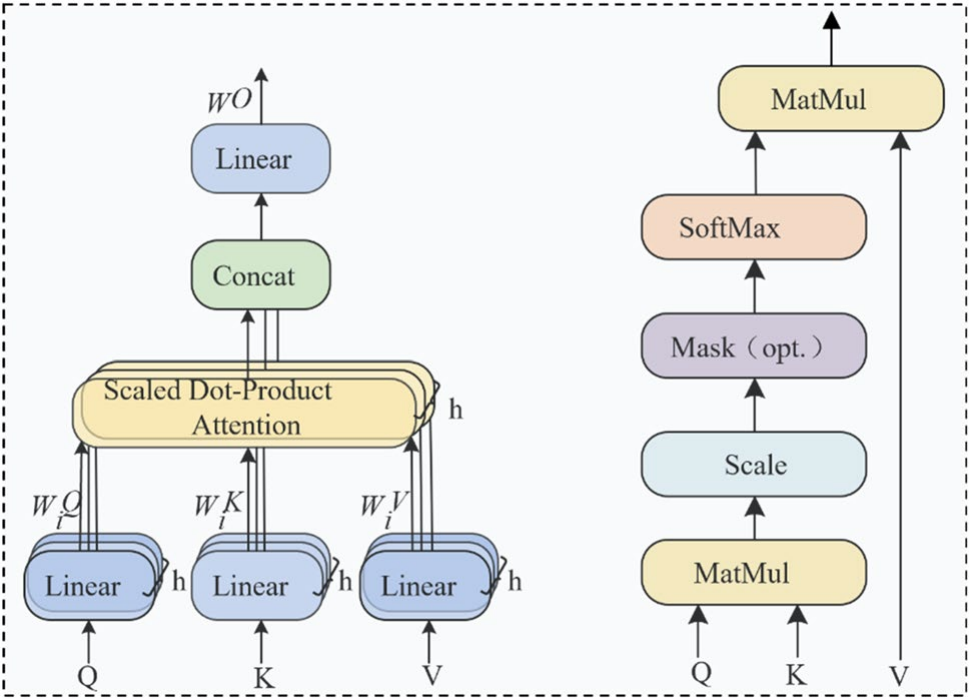
Multiple head scaling point product attention structure.

The improved Transformer model retains the multi-head scaled dot-product attention mechanism from the original Transformer. This attention mechanism involves mapping a query (*Q*) and a set of key-value pairs (*K*-*V*) to generate an output. The output is calculated as the weighted sum of values (*V*), with the weights determined by the similarity between the query and each value. The outputs of h scaled dot-product attentions are then fused together to generate the final output, where each attention output is referred to as a head. As shown in Fig. 2, it illustrates the structure of the multi-head scaled dot-product attention.

(1) Scaled Dot-Product Attention.

1 $$Attention(Q,K,V)=soft\mathit{max}\left(\frac{Q{K}^{T}}{\sqrt{{d}_{k}}}\right)V$$

(2) Multi-Head Scaled Dot-Product Attention.

2 $$MultiHead(Q,K,V)=Concat(hea{d}_{1},\dots ,head{\hspace{0.33em}}_{h}){W}^{O}$$

3 $$where\hspace{0.33em}head{d}_{i}=Attention\left(Q{W}_{i}^{Q},K{W}_{i}^{K},V{W}_{i}^{V}\right)$$

where *W*_i_^Q^ ∈ R^dmodel×dk^, *W*_i_^K^ ∈ R^dmodel×dk^, *W*_i_^V^ ∈ R^dmodel×dk^, *W*^O^ ∈ R^hdv^ × d_model_, *h* represents the number of attention heads, *d*_k_ = d_model_/h, d denotes the dimension of the vectors, and T denotes the transpose of a matrix.

### RS-LSTM-transformer

**Figure 3:**
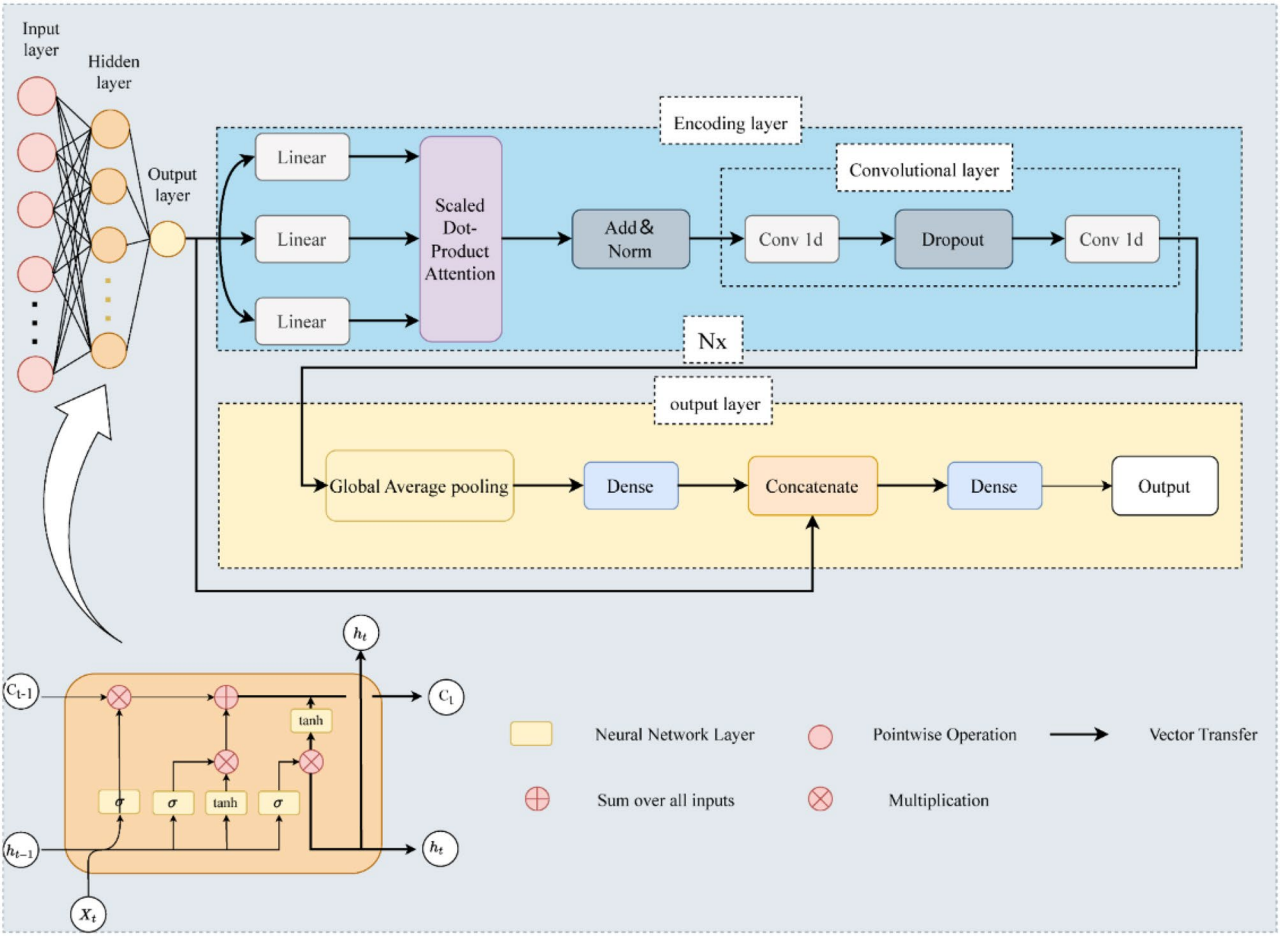
The structural diagram of the hybrid model combining LSTM and the Transformer with improved architecture.

In our improved Transformer model, we had integrated an LSTM layer in the input section to facilitate feature extraction and reconstruction of time series data. The LSTM layer effectively utilizes current data features and leverages its gate mechanism to determine whether to retain or forget previous features. As illustrated in Fig. 3, the integration leads to the establishment of an LSTM-Transformer model. The LSTM-Transformer model consists of several components: a single-layer LSTM with hidden units, multiple encoding layers (Encoders), and an output layer. The encoding layers include a multi-head scaled dot-product Attention, residual connections (Add), normalization (Norm), and convolutional layers with two one-dimensional convolutions (Conv1d). The convolutional layers are utilized to extract deep features from the data, reducing the number of trainable parameters through weight sharing and sparse connections, thereby enhancing forward propagation efficiency. Additionally, a Dropout layer is incorporated to prevent overfitting of the model. The output layer incorporates a Global Average Pooling layer to transform the vectorized data into a one-dimensional representation by taking the average across all dimensions. Subsequently, the data passes through a Dense layer for the final output. Finally, the output results are concatenated with the LSTM layer and passed through another Dense layer for the ultimate output. We employed the RS optimization algorithm to construct the optimal RS-LSTM-Transformer model with the best parameters.

## Case study

As one of the largest rivers in China, the Yellow River has a vast watershed with complex and variable hydrological characteristics. Factors such as climate change have made flooding in the Yellow River a complex and serious problem. In particular, there have been significant changes in the underlying surface properties in the middle reaches of the Yellow River in the past 50 years.

**Figure 4:**
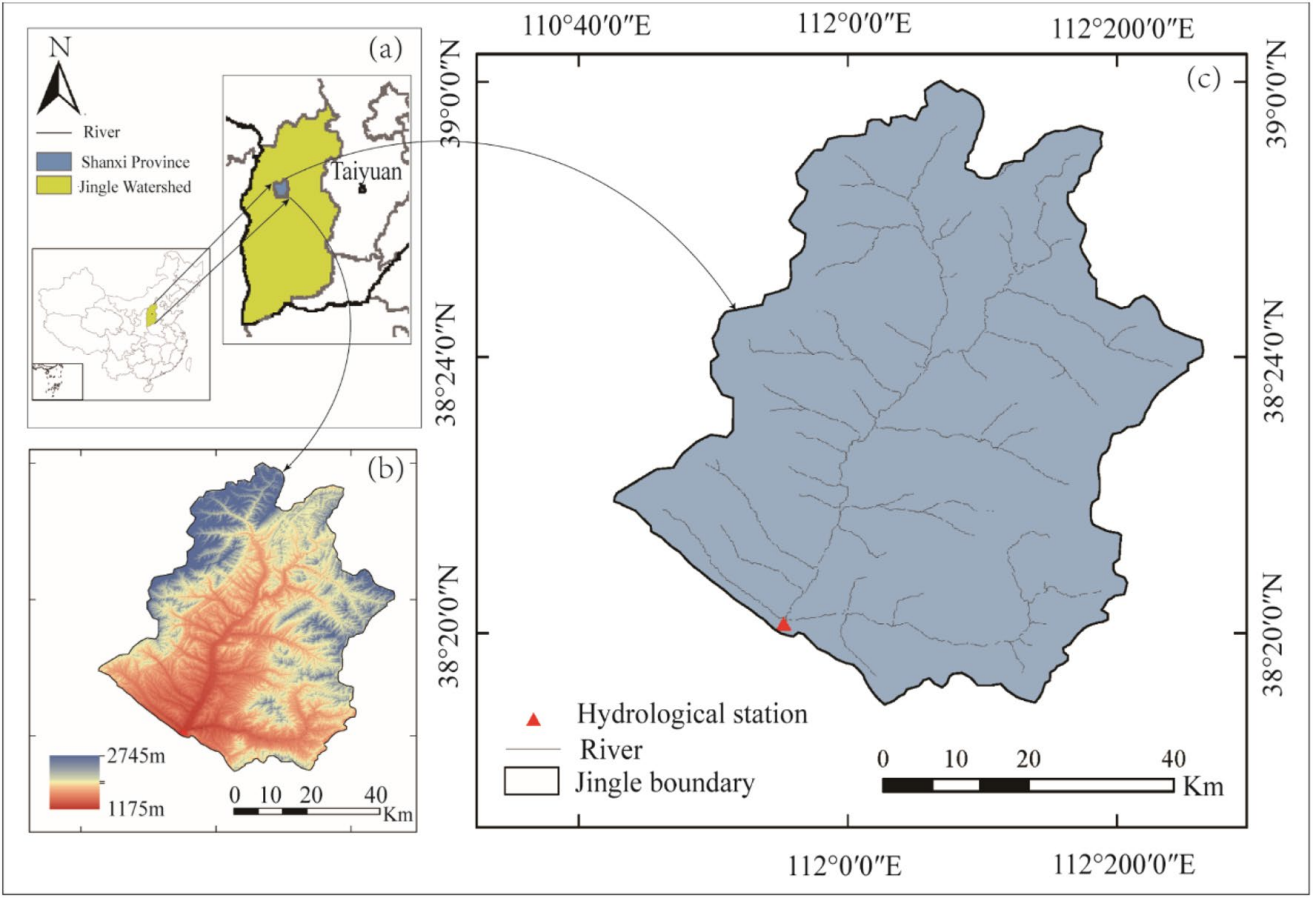
The position and station distribution, as well as the river water system distribution, in the Jingle watershed in the middle reaches of the Yellow River, (**a**) depicts the location of the watershed, (**b**) illustrates the topographic variations within the watershed, while (**c**) presents a detailed hydrological network of the watershed (The map was created using ArcMap 10.8 software, and the drawing boundaries are sourced from https://www.gpsov.com/cn2/).

As illustrated in Fig. 4, the Jingle watershed was selected as the representative research area for this study. Located in the northwest of Shanxi Province, China, the Jingle watershed is the second-largest tributary of the Yellow River in its middle reaches. The Jingle River originates from Shenchixian County, Xinzhou City, Shanxi Province, with a total length of 83.9 km. The average slope of the main stream is 6.7‰, and the watershed area is 2799 km^2^, passing through Ningwuxian County and Jinglexian County in Shanxi Province. The Jingle watershed is located in the Loess Plateau region of the middle reaches of the Yellow River and has a semi-humid and semi-arid continental monsoon climate. The average annual temperature in the watershed ranges from 3 °C to 12 °C, decreasing from south to north. The average annual precipitation is 538 mm, with large interannual variations and uneven spatial distribution. The average annual maximum peak discharge is 596 m^3^/s, with the measured maximum peak discharge reaching 2267 m^3^/s. The Jingle watershed is one of the areas prone to flood disasters in the middle reaches of the Yellow River.

The Jingle Hydrological Station, serving as the control station for the Jingle watershed, is located at approximately 111°55' east longitude and 38°20' north latitude. In this study, we collected hourly flow data from the Jingle Hydrological Station from 1971 to 2013, as well as hourly rainfall data from 14 other stations. These data cover the complete processes of 98 flood events. Among them, the first 78 floods were used for model calibration, while the remaining 20 floods were used for model validation. As shown in Table 1, the statistical characteristics of 98 flood events in the Jingle River watershed are detailed, with key features including Total Rainfall, Rainfall Duration, Rainfall Center, and Peak Discharge. The data indicate significant variations in rainfall duration, peak discharge, and rainfall center among events, highlighting the complexity of the rainfall-runoff process and the challenges in effectively modeling it.

**Table 1:** Statistical Characteristics of 98 Flood Events in the Jingle River watershed, 1971–2013.

Flood events	Initial date	Total rainfall (mm)	Rainfall duration (h)	Rainfall center	Peak discharge (m^3^/s)
1	1 July 1971	8.86	36	Ninghuabao	164.50
2	23 July 1971	63.40	69	Chunjingwa	261.21
3	31 July 1971	10.44	12	Dongzhai	286.00
4	7 August 1971	21.07	42	Ninghuabao	184.14
5	15 August 1971	7.60	16	Chunjingwa	145.00
6	27 August 1971	15.71	36	Chunjingwa	112.00
…	…	…	…	…	…
93	23 September 2008	70.49	88	Oidongzi	132.00
94	10 August 2010	70.50	24	Songjiaya	67.00
95	11 July 2011	41.88	24	Dujiacun	54.35
96	26 July 2012	40.57	41	Ninghuabao	134.00
97	30 July 2012	41.95	41	Chashang	61.90
98	17 July 2013	29.91	32	Jingle	74.40

## Experiments

### Model environment settings and data processing

The experiments conducted, encompassing the current and subsequent ones, were carried out within the parameters outlined in Table 2.

**Table 2:** The environment.

CPU	GPU	RAM
AMD Ryzen 7 4800H	NVIDIA RTX 3070 8 GB	16 GB
OS	Complier	Library
Widows 10 × 6	Python 3.7.10	Tensorflow 2.4.0

First, we preprocessed the flood element data into a sliding window format and define an appropriate time step. By sliding the window along the time series, we extracted the data within each window as input features for the model. He input flood data can be represented as ***U*** = [*Q*,*P*], where the runoff data matrix is ***Q*** = [*Q*(t-*T*-*i*), *Q*(*t*-*T*-*i* + 1),… *Q*(*t*-*T*)]^T^, and the rainfall data matrix is ***P*** = [*P*_1_,* P*_2_,…,*P*_13_], ***P***_**j**_ = [*p*_j_(*t*-*T*-i), *p*_j_(*t*-*T*-i + 1),…, *p*_j_(*t*-*T*)]^T^, (*j* = 1,2,…,13)(*T* represents the lead time, *i* denotes the time step) , The input shape is [(None,i,14)]. Here, *Q*(*t*-*T*) represents the discharge at the current time, and *Q*(*t*-*T*-*i*) represents the discharge at historical times;* p*_1_(*t*-*T*),* p*_2_(*t*-*T*), …, *p*_j_(*t*-*T*) represent the precipitation at the first to *j-th* rainfall stations.

Next, we normalized the data using the max–min normalization method, scaling the values to fit within the range of (0,1). This normalization process helps improve the convergence speed and stability of the deep learning model, mitigates gradient propagation issues, enhances the generalization capability of the model, and optimizes the parameter updating process. Finally, we applied inverse scaling to the predicted data from the model to restore their physical meaning and interpretability, obtaining the final predicted discharge results.

### RS-LSTM-transformer model building process

In the flood forecasting process, we employed a sliding window approach to perform rolling forecasts of runoff at different lead times. The sliding window moves along the time axis until it reaches the end of the dataset. The input variables consist of antecedent rainfall and runoff features. The LSTM-Transformer flood forecasting model is utilized to capture the nonlinear relationship between rainfall and runoff, and the RS optimization algorithm is employed to construct an optimal LSTM-Transformer model with the best parameters, resulting in the final output forecast.

**Figure 5:**
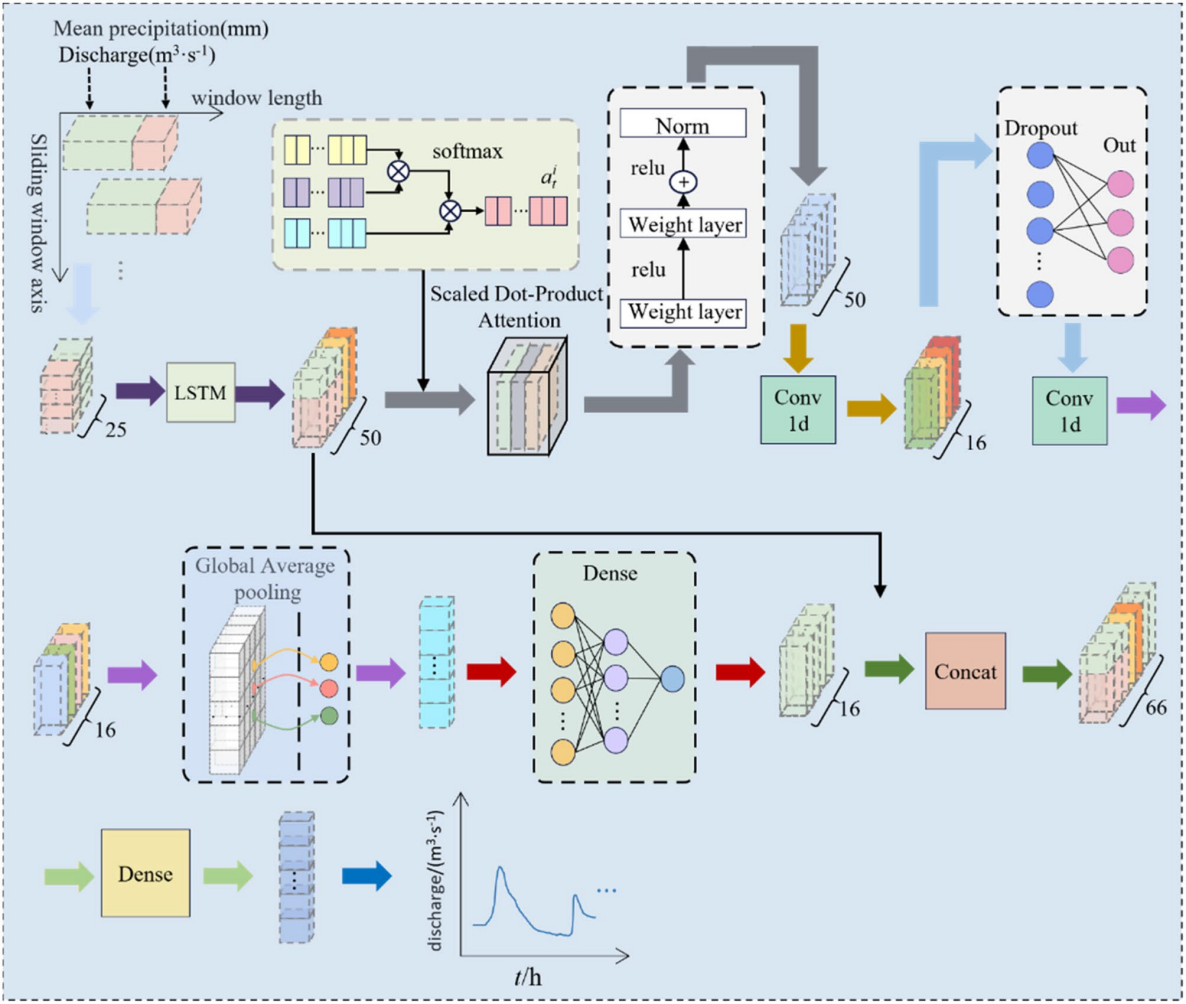
Detailed schematic diagram of the LSTM-Transformer flood forecasting model.

As shown in Fig. 5, it illustrates the detailed schematic diagram of the LSTM-Transformer flood forecasting model, depicting the prediction process. Equations (4) and (5) represent the format of the input and output data for the model, and the specific process from input to output is as follows:

1. Data Input: The flood input data is represented as ***U*** = [*Q*, *P*].
2. LSTM Layer: The LSTM layer incorporates additional data features ***O*** and produces the vector ***U***_***G***_ = [*Q*_G_, *P*_G_, *O*_G_]; The output shape is [(None, 1, 50)].
3. Multi-Head Attention Layer: The multi-head attention layer maps the input to multiple subspaces. The output shape is [(None,1,50)].
4. Residual & Normalization: The original input is added to the output from the previous step to prevent gradient explosion. The output shape is [(None,1,50)].
5. Convolutional Layers and Dropout Layer: Instead of fully connected layers, two one-dimensional convolutional layers are utilized for non-linear mapping. Dropout is applied to mitigate overfitting. The output shape is [(None,1,50)].
6. Global Average Pooling and Fully Connected Layer: Global average pooling reduces the dimensionality of the data, while regularization is applied to the entire network structure to prevent overfitting. The output is obtained through a fully connected layer. The output shape is [(None,1,16)].
7. Concatenate Feature Fusion and Fully Connected Layer: The output from the LSTM layer is concatenated with the output from the previous step, and the final result is obtained through a fully connected layer. The output shape is [(None,1)].
  4 $$U = \left( {\begin{array}{*{20}c} {Q\left( {t - T - i} \right)} & {p_{1} \left( {t - T - i} \right)} & \cdots & {p_{13} \left( {t - T - i} \right)} \\ {Q\left( {t - T - i + 1} \right)} & {p_{1} \left( {t - T - i + 1} \right)} & \cdots & {p_{13} \left( {t - T - i + 1} \right)} \\ \vdots & \vdots & \ddots & \vdots \\ {Q\left( {t - T} \right)} & {p_{1} \left( {t - T} \right)} & \cdots & {p_{13} \left( {t - T} \right)} \\ \end{array} } \right)$$
  5 $$Q_{{{\text{output}}}} = \left( \begin{gathered} Q\left( T \right) \hfill \\ Q\left( {T + 1} \right) \hfill \\ \vdots \vdots \vdots \vdots \vdots \vdots \hfill \\ Q\left( {T + {\text{n}}} \right) \hfill \\ \end{gathered} \right)$$

### Model parameters comparison and optimization

Before training the model, it is crucial to establish appropriate hyperparameters based on experimental settings^62^. Through preliminary experiments, we identified five parameters that significantly influence the training performance of the LSTM-Transformer model: attention head dimension, number of heads, fully connected layer dimension, number of encoding blocks, and LSTM layer neuron count. For comparative analysis, we also included the LSTM, Transformer, BP, and MLP models, utilizing *NSE* as the function. The RS algorithm was employed to identify the best hyperparameters. To enhance training efficiency, we focused on discussing a subset of key parameters. As shown in Table 3, it displays the parameter configurations for each model. In the RS algorithm, we set the number of random samples (n_iter) to 50, the number of cross-validation folds (cv) to 5, and the random seed (random_state) to 5. By employing these settings, we successfully determined the optimal combination of hyperparameters for the LSTM-Transformer model, resulting in exceptional performance.

**Table 3:** Model parameter setting.

\	LSTM-transformer	LSTM	Transformer	BP	MLP
Common parameters	Number of heads = [8256] Number of cells = [16,256]	Number of cells = [16,256]	Number of cells = [16,256]	Number of cells = [16,256]	Number of cells = [16,256]
Shared parameters	Time steps = [1, 12] Batch size = [24,128] Learning rate = 0.001 Dropout = 0.1				
Other parameters	Number of encoding blocks = 4 Attention head dimension = 32 Fully connected layer dimension = 64	Number of hidden layers = 1	Number of encoding blocks = 4 Attention head dimension = 64 Fully connected layer dimension = 64	Number of hidden layers = 1	Number of hidden layers = 2

### Comparison between different model benchmarks

To comprehensively evaluate the performance of the LSTM-Transformer model, we selected LSTM, Transformer, BP, and MLP as benchmark models. These models have been widely utilized in time series forecasting and extensively studied and validated. To ensure fairness and reliability, the RS algorithm was employed to optimize the hyperparameters of these benchmark models. During the comparison analysis, both the benchmark models and the LSTM-Transformer model were assessed at identical lead times (T = 1, 2, 3, 4, 5, 6 h). Evaluation metrics were calculated for all models to quantify their prediction accuracy, stability, and fitting capability.

LSTM is a specialized type of RNN that overcomes the gradient vanishing problem and effectively addresses long-term nonlinear dependencies^41^. In the field of hydrology, flood processes often exhibit complex nonlinear and time-varying characteristics. The memory cells and forget gates within LSTM enable the model to automatically select and retain important historical information, thereby enhancing the accuracy and reliability of flood discharge forecasting. Gated Recurrent Unit (GRU) models, possessing a structure akin to LSTM but with less complex computations and a reduced number of parameters, are employed as benchmark models as well^63^. Therefore, LSTM and GRU are used as benchmark models.

Among all Artificial Neural Networks (ANNs), Backpropagation Neural Network (BP) is a powerful algorithm widely applied in flood forecasting^64^. Based on the error backpropagation algorithm, the BP model is a feedforward neural network that can capture nonlinear relationships and temporal features in flood processes through training and learning from historical flood data^65^. Therefore, BP is employed as a benchmark model.

Multilayer Perceptron (MLP) models are feedforward neural networks with multiple hidden layers capable of effectively handling nonlinear problems^66^. Flood processes are influenced by complex interactions among multiple factors, and the combination of multiple hidden layers and the introduction of nonlinear activation functions in MLP models allow for better capturing of these complex relationships^67^. Hence, MLP is also employed as a benchmark model.

### Performance evaluation criteria

In this study, the performance of the model's predictions was evaluated using the Nash Sutcliffe efficiency (*NSE*), root mean square error (*RMSE*), mean absolute error (*MAE*), and bias as evaluation metrics. The mathematical expressions for these metrics are as follows:

6 $${ }NSE = 1 - \frac{{\mathop \sum \nolimits_{i = 1}^{n} \left( {Q - Q_{i}^{{}} } \right)^{2} }}{{\mathop \sum \nolimits_{i = 1}^{n} \left( {Q - \overline{Q}_{i} } \right)^{2} }}$$

7 $$RMSE = \sqrt {\frac{{\sum\limits_{i = 1}^{n} \; (Q - Q_{{\text{i}}}^{{}} )^{2} }}{n}}$$

8 $$MAE = \frac{{\sum\limits_{i = 1}^{n} {|Q_{{\text{i}}}^{{}} - Q|} }}{n}$$

9 $$B{\text{ias}} = \frac{{\mathop \sum \nolimits_{i = 1}^{n} \left( {Q_{i}^{{}} - Q} \right)}}{{\mathop \sum \nolimits_{i = 1}^{n} \left( Q \right)}}$$

In the equations, $Q$ and $Q_{i}^{{}}$ the discharge of the simulated and observed hydrographs, respectively.$\overline{Q}$ and $\overline{Q}_{i}^{{}}$ represent the mean of the discharge of the simulated and observed hydrographs, respectively. *i* represents the *i-th* moment, n is the data points number;

NSE values range from − ∞ (no fit) to 1 (perfect fit); RMSE spans from 0 (perfect fit) to + ∞ (no fit). MAE, the average absolute error, describes the difference between observed data and simulation outcomes. Bias measures the deviation of predictions from actual values, with a range from − 100% to 100%, where closer to 0 indicates smaller deviations^53^. However, these model indicators have limitations; for example, RMSE quantifies error size but is overly sensitive to large errors, obscuring the model's normal performance. MAE indicates average error levels but does not distinguish error direction, lacking the ability to display model biases. Therefore, model evaluations should integrate multiple indicators such as NSE, RMSE, MAE, and Bias for a comprehensive performance analysis^68^.

## Results and discussion

### Comparison of overall flood forecasting effects

**Figure 6:**
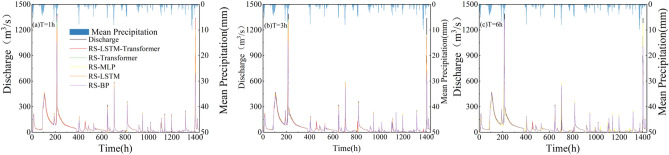
Comparison of overall flood forecasting results between the calibration period and validation period for five models within different lead times of 1, 3, and 6 h.

As illustrated in Fig. 6, it presents observed and estimated hydrographs of the RS-LSTM-Transformer, RS-LSTM, RS-Transformer, RS-BP, and RS-MLP models, representing five different models, during the calibration and validation periods for lead times of 1h, 3h, and 6h. It can be observed that the predicted discharge hydrographs closely match the observed discharge hydrographs.

As shown in Table 4, it presents a statistical evaluation of the forecasting performance of the five models at lead times of 1h, 3h, and 6h. Upon comparing the results, it is clear that the RS-LSTM-Transformer model exhibits the best forecasting performance at a lead time of 1h. At 1h of lead time, the NSE, RMSE, MAE, and Bias% during the calibration and validation periods are 0.970, 14.001 m^3^/s, 5.304 m^3^/s, 0.501% and 0.953, 14.124 m^3^/s, 6.365 m^3^/s, 0.523%, respectively, indicating that the predictions are very close to actual values. At 6h of lead time, the accuracy of the RS-LSTM-Transformer model decreases; the corresponding values for NSE, RMSE, MAE, and Bias% during the calibration and validation periods are 0.892, 35.522 m^3^/s, 24.828 m^3^/s, 8.896% and 0.875, 35.674 m^3^/s, 26.677 m^3^/s, 9.958%. Compared to the RS-LSTM, RS-Transformer, RS-BP, and RS-MLP models, the RS-LSTM-Transformer model consistently outperforms them in terms of various forecasting performance evaluation metrics. This demonstrates its superior capability in capturing the non-linear relationship between rainfall and runoff. As shown in Supplementary Tables 1 and 2, the performance of each model under initial parameter settings and trial-and-error tuning methods is displayed. A comparison with Table 4 reveals the clear superiority of using the RS optimization algorithm for hyperparameter tuning across models, as opposed to initial settings and random trial-and-error methods.

**Table 4:** Performance comparison of five models in runoff prediction during calibration and validation periods (Lead times = 1 h, 3 h, and 6 h).

Lead time(h)	Model	Calibration				Validation			
		NSE	RMSE/(m^3^·s^−1^)	MAE/(m^3^·s^−1^)	Bias%	NSE	RMSE/(m^3^·s^−1^)	MAE/(m^3^·s^−1^)	Bias%
1 h	RS-MLP	0.909	25.574	16.852	1.130	0.908	26.323	17.321	1.144
	RS-BP	0.905	28.012	18.012	0.970	0.901	28.125	18.311	0.972
	RS-Transformer	0.925	18.327	10.147	0.781	0.920	19.524	11.128	0.816
	RS-LSTM	0.949	15.745	8.393	0.615	0.943	16.125	8.669	0.681
	RS-LSTM -Transformer	0.970	14.001	5.304	0.501	0.953	14.124	6.365	0.523
3 h	RS-MLP	0.865	34.102	27.382	7.101	0.861	39.102	28.312	7.124
	RS-BP	0.872	35.524	27.008	7.401	0.862	36.174	28.705	7.512
	RS-Transformer	0.901	27.058	19.037	5.982	0.882	28.211	20.155	6.332
	RS-LSTM	0.926	21.001	14.005	5.605	0.915	21.358	14.053	5.580
	RS-LSTM -Transformer	0.948	18.024	10.854	4.072	0.932	19.108	10.633	4.105
6 h	RS-MLP	0.761	78.285	63.757	21.857	0.751	80.124	64.135	22.151
	RS-BP	0.788	71.207	59.278	20.001	0.783	72.258	61.117	20.855
	RS-Transformer	0.843	52.570	43.877	12.195	0.832	55.845	45.844	14.102
	RS-LSTM	0.861	42.003	33.005	11.003	0.859	42.102	33.185	11.384
	RS-LSTM -Transformer	0.892	32.522	24.828	8.896	0.875	35.674	26.677	9.958

**Figure 7:**
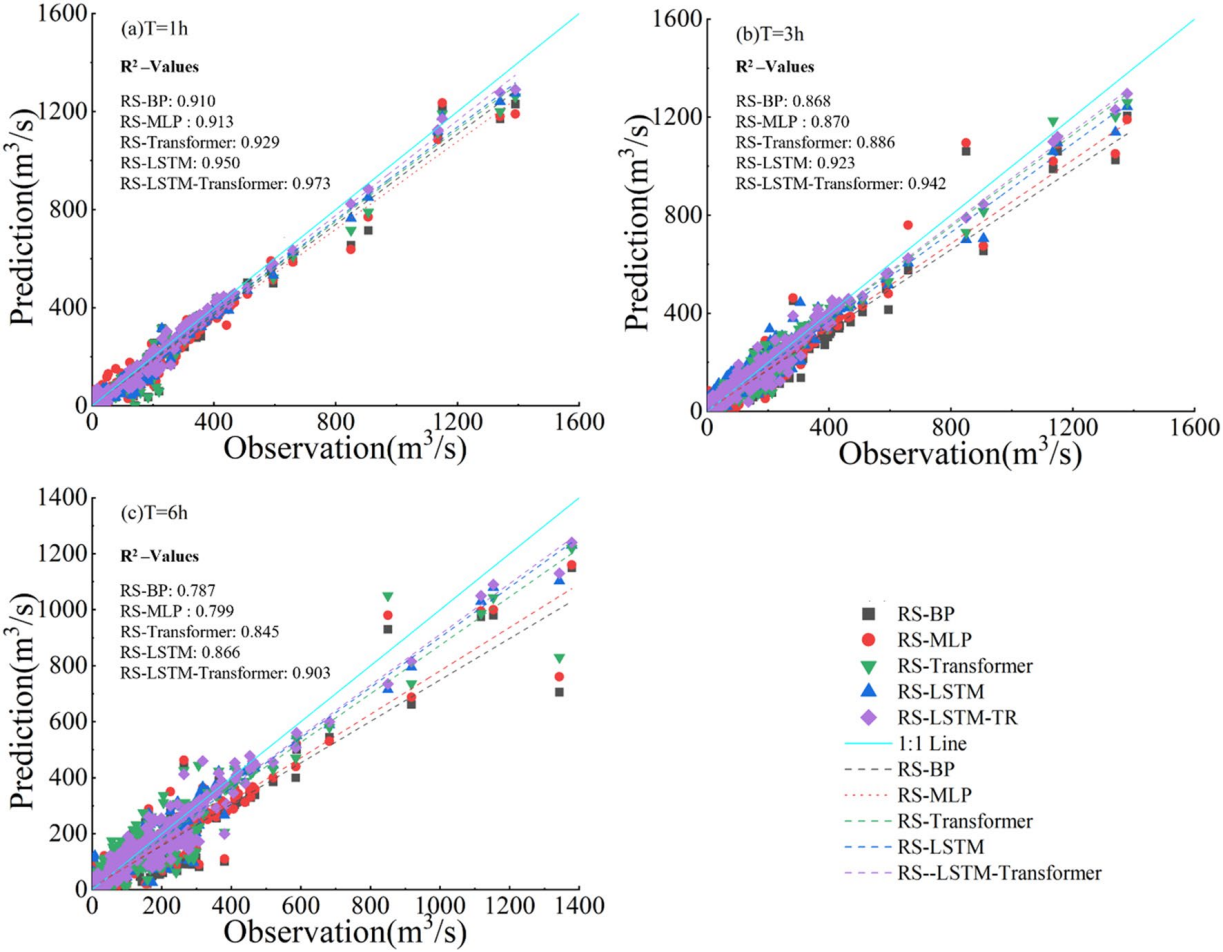
Scatter plots of observed and predicted discharge under different lead times for five models in the validation period.

As shown in Fig. 7, The scatter plots in Fig. 7 compare the observed and predicted values for different lead times (T = 1 h, 3 h and 6 h) during five model validation periods. It can be observed that compared to the RS-LSTM, RS-Transformer, RS-BP, and RS-MLP models, the scatter plots and fitting lines of the RS-LSTM-Transformer hybrid model are closer to the 1:1 line. This indicates that the RS-LSTM-Transformer model has smaller deviations between predicted and observed values, outperforming other models at the same lead time and better reflecting the relationship between predicted and observed discharge. Therefore, the performance of the RS-LSTM-Transformer model in runoff forecasting is superior to that of other models.

However, each model shows several outliers at T = 3 and T = 6 h, mostly centered at high discharge points. By comparing the model-predicted discharge points with the observed values, two reasons for this phenomenon were identified: firstly, the models exhibit abnormal responses under the influence of sudden changes in rainfall and runoff data; secondly, as the lead time increases, the model predictions show a lagging effect, resulting in a significant discrepancy between the lagged high discharge points and the contemporaneous observed discharge, which is manifested as outliers in the graph.

Moreover, a clear observation from Fig. 7 and Table 4 reveals that the accuracy of predictions from all five models decreases as the lead time increases. From panels (a), (b), to (c) of Fig. 7, the correlation coefficient (R^2^) for the RS-LSTM-Transformer model is 0.973, 0.942, and 0.903 at lead times of 1 h, 3 h, and 6 h, respectively. The data points predicted by each model, including the RS-LSTM-Transformer, are more dispersed. This is due to a larger time interval between the inputs and outputs in the training set reduces the data correlation, leading to decreased prediction accuracy of machine learning models.

**Figure 8:**
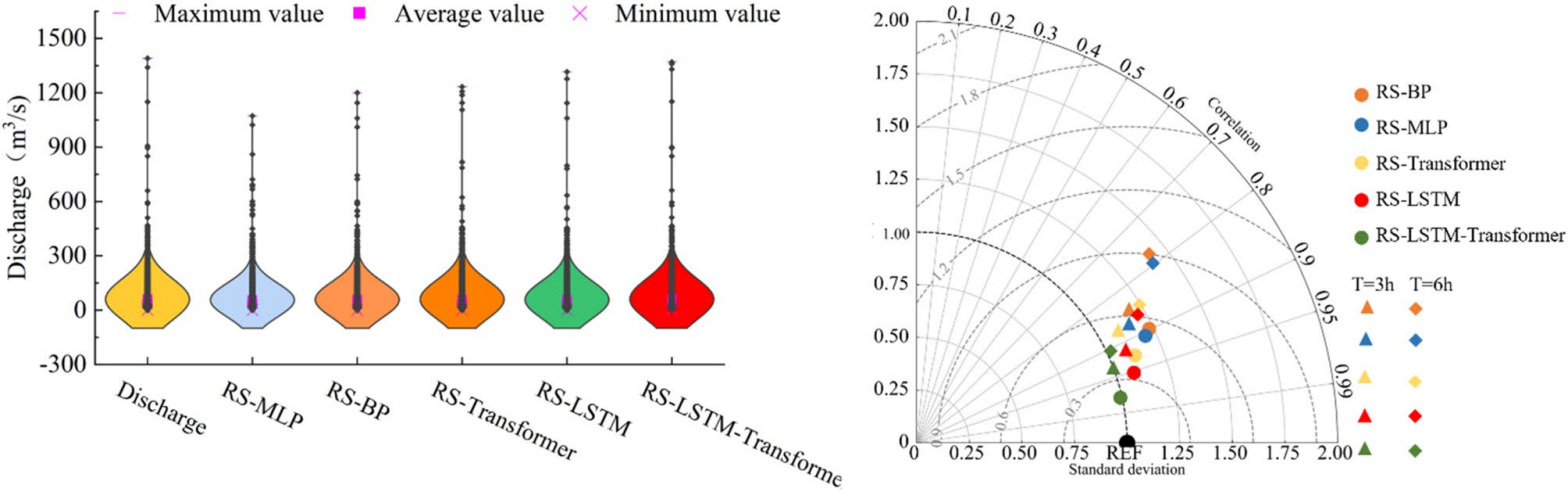
Comparison of Taylor Diagrams and Violin Plots for Various Models During the Validation Period at Jingle Station.

As depicted in Fig. 8, the Taylor diagrams and violin plots compare various models during the validation period at Jingle Station. In panels 8(a) and 8(b), the implemented models are further compared using Taylor diagrams and violin plots. The Taylor diagrams clearly show that the RS-LSTM-Transformer model has a standard deviation closest to the observed values, the highest correlation, and the lowest squared errors, followed by the RS-LSTM and RS-Transformer models. The violin plots vividly demonstrate that the distribution of predicted flow points by the RS-LSTM-Transformer model is closest to the actual measurements, while the RS-MLP model’s distribution is the most divergent, especially visible in the distribution of predicted peak values. All these charts validate the test statistics provided in Table 4, indicating that the RS-LSTM-Transformer model outperforms others in short-term flood forecasting tasks.

### Comparison of single-event flood forecasting effectiveness

**Figure 9:**
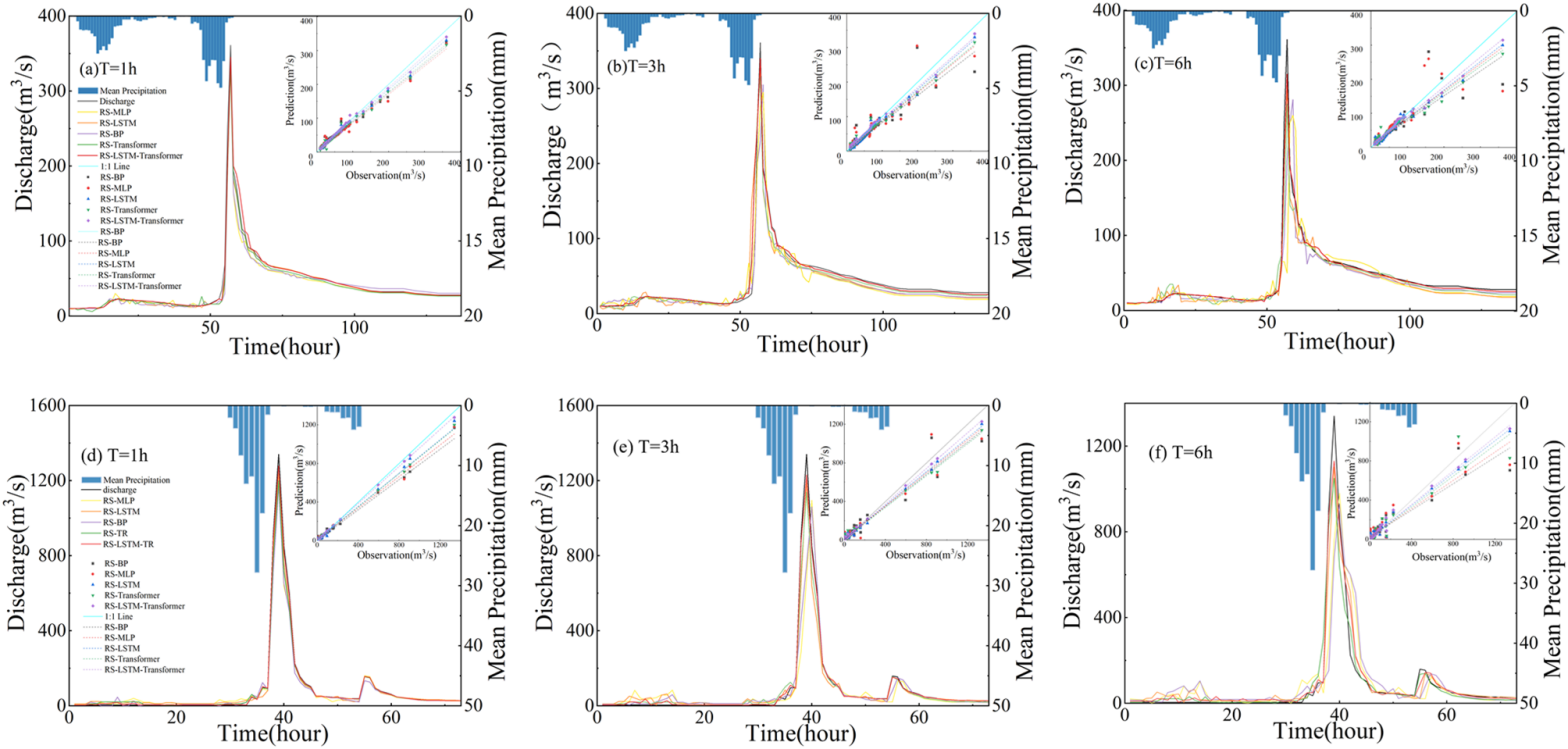
Comparative analysis of observed discharge and prediction results of RS-LSTM-Transformer, RS-LSTM, RS-Transformer, RS-BP, and RS-MLP models (flood 1 : event 19,980,711, flood 2: event 20,030,729).

As shown in Fig. 8, in order to evaluate the performance of five models in flood forecasting, we conducted model validation on two typical flood events during the verification period. The two events, referred to as Flood 1 (Fig. 9a–c) and Flood 2 (Fig. 9d–f), represent different characteristics. Flood 1 is a single-peak flood event with a relatively low peak discharge, while Flood 2 is a double-peak flood event with a higher peak discharge. By comparing the rainfall-runoff hydrographs and scatter plots in Fig. 9, we further analyzed the differences in the model predictions for these two flood events at different lead times (T = 1 h, T = 3 h and T = 6 h), aiming to gain deeper insights into the performance of different models. The dispersion of data points in the scatter plots and the distance between the fitted line and the 1:1 line in Fig. 9 provide visual indications of the magnitude of errors between the observed and predicted discharge under different lead times and different models.

Based on the observation of the rainfall-runoff graph in Fig. 9a, it can be observed that when the lead time is 1 h, the predicted flow process lines of the five models closely match the measured flow process line. However, there are some fluctuations in the RS-MLP and RS-BP models during the lower discharge stages before the peak discharge. In comparison, the RS-Transformer and RS-LSTM models perform better, with the RS-LSTM-Transformer model exhibiting the best stability. At lead times of 3 h and 6 h (Fig. 9b,c), the predictive performance of all five models decreases, and the differences gradually become apparent.

We primarily analyzed these five models in terms of the stability, lagging effect, and underestimation of peak discharge in predicted results. From a stability perspective, we compared the fluctuation levels of the simulation results of the RS-MLP and RS-BP models to the observed values. We found that when the lead time increased from 1 to 3 h, the fluctuation level of these two models also increased. Additionally, when the lead time was extended to 6 h, both models exhibited significant fluctuations before and after the peak discharge. The RS-Transformer model also showed fluctuations under a 6-h lead time. In comparison, the RS-LSTM and RS-LSTM-Transformer models have largely overcome this issue, with their predicted flood hydrographs displaying minor fluctuations but overall exhibiting stable performance^69^. However, when the lead time reached 6 h, the performance of the RS-LSTM model was inferior to that of the RS-LSTM-Transformer model. Regarding the lagging effect in the models' prediction results, the RS-Transformer, RS-LSTM, and RS-LSTM-Transformer models performed well, while the other two models showed noticeable lag. Concerning the underestimation of peak discharge, the predicted values of all models were generally lower than the observed values in most flood forecasting processes. However, the RS-LSTM-Transformer model more accurately predicted the peak discharge compared to the LSTM model, showing closer agreement with the actual flood process. This suggests that the RS-LSTM-Transformer model is more sensitive to rainfall and runoff processes.

As the lead time increases, the forecasting performance of all models deteriorates, and there is an increasing tendency to underestimate peak discharge and lag. This is because as the lead time lengthens, the correlation between the input data and the target discharge data decreases, making it more challenging to accurately learn and extract flood data features. Compared to other models, the RS-LSTM-Transformer model exhibits smaller overall prediction bias at different lead times and achieves the best performance in flood forecasting and rainfall-runoff simulation.

Additionally, when comparing the prediction results of two flood events with different characteristics (single-peak shape and lower peak discharge vs double-peak shape and higher peak discharge) at lead times of 1, 3, and 6 h, as shown in Fig. 9a–f, all models perform worse in simulating the flood event with a double-peak shape and higher peak discharge. This is due to the involvement of complex nonlinear and spatiotemporal variations in the rainfall-runoff process. For the flood event with a double-peak shape and higher peak discharge, its formation mechanism may involve more environmental factors and hydrological processes, introducing more uncertainty and stochastic factors that make its characteristics harder to capture by current models. From the predicted results of the peak discharge for Flood Event 1 (slightly below 500 m^3^/s) and the two peaks in Flood Event 2 (one slightly above 1200 m^3^/s and the other slightly below 200 m^3^/s), it can be observed that the RS-MLP model is more sensitive to high-flow flood processes, while the RS-BP model provides more accurate predictions for low-flow peak floods.

Finally, comparing the performances of RS-Transformer and RS-LSTM in terms of peak magnitude and lag, it is found that LSTM can more accurately predict the peak magnitude, while the Transformer model is closer to the actual situation in terms of lag. When employing machine learning in flood forecasting models, it is common to encounter challenges such as the underestimation of peak flows and delays in forecasts. Previous studies have identified these issues with single models like LSTM in flood prediction tasks^70^. However, the hybrid RS-LSTM-Transformer model has markedly improved these shortcomings. The RS-LSTM-Transformer model effectively combines the features of both LSTM and Transformer. LSTM excels at capturing long-term dependencies and temporal patterns within sequences, while Transformer enhances the model's ability to discern dependencies between different positions in the sequence. Consequently, the RS-LSTM-Transformer model more accurately captures temporal patterns and dependencies in flood flow forecasting, leading to improved predictive performance. Therefore, the RS-LSTM-Transformer model can more accurately capture the temporal patterns and dependencies in flood discharge forecasting, thereby improving the prediction performance.

### Model performance evaluation

#### Robustness evaluation

**Figure 10:**
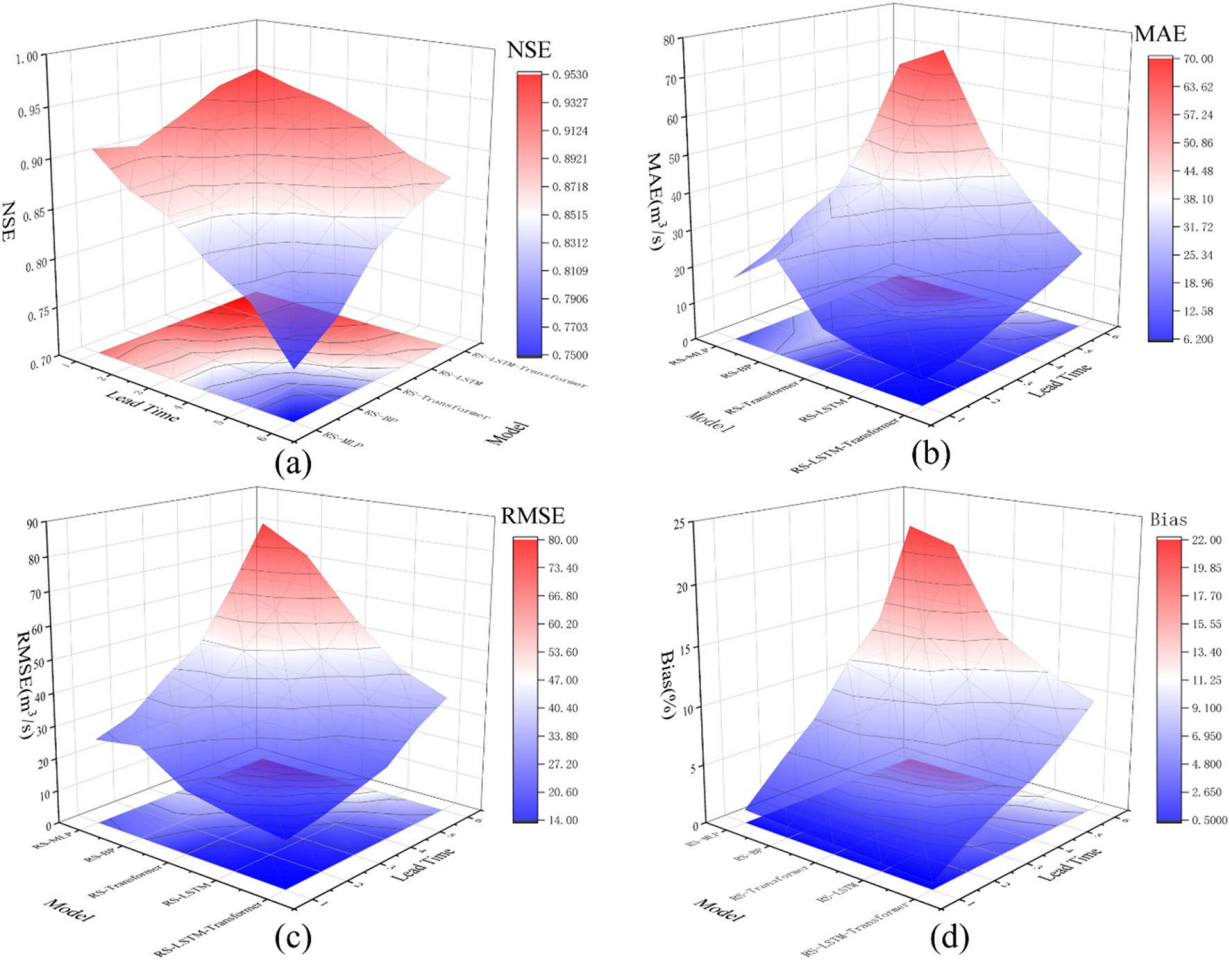
Robustness evaluation results of RS-LSTM-Transformer, RS-LSTM, RS-Transformer, RS-BP, and RS-MLP models.

Four evaluation metrics, namely *NSE*, *MAE*, *RMSE*, and *Bias*, were employed in the study to evaluate the predictive performance of the models. As shown in Fig. 10, it illustrates the trends of these evaluation metrics during the validation period, with model type and lead time as the x–y axes and the model evaluation metrics as the z-axis.

Comparative analysis through subfigures 10(a), (b), (c), and (d) clearly demonstrates that under the same forecast horizons, the RS-LSTM-Transformer model excels across all evaluation metrics—NSE, MAE, RMSE, and Bias—highlighting its superior performance in simulating rainfall-runoff. The predictive accuracy of all five models declines as the lead time extends, illustrating the impact of lead time on model precision. Figure 10 collectively showcases the higher robustness of the RS-LSTM-Transformer model in long-term forecasting. Although its accuracy decreases over time, the decline is relatively gradual. In contrast, the RS-BP and RS-MLP models display a steeper drop in performance indicators, particularly when the lead time period exceeds four hours, as dramatically evident in subfigure 10(d), underscoring their limitations in long-term predictions. The development of the RS-LSTM-Transformer model represents a novel endeavor in the field of flood forecasting using deep learning, showcasing its effectiveness in flood prediction and simulation. In the future, adjustments to the model structure to reduce training time and the incorporation of supplementary methods should be considered to enhance both the accuracy and stability of the predictions.

#### Comparison of model training efficiency

**Figure 11:**
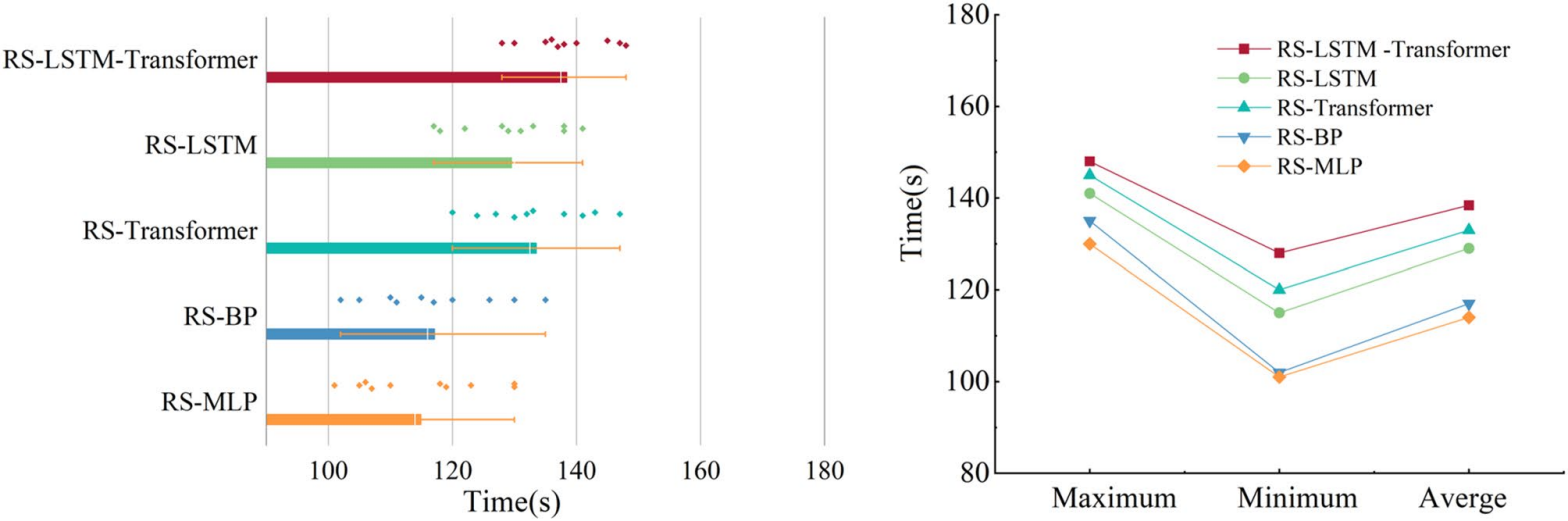
Comparison of Training Efficiency and Time Consumption for LSTM-Transformer, LSTM, Transformer, BP, and MLP Models.

As shown in Fig. 11, it presents the training time statistics for five models (RS-LSTM-Transformer, RS-LSTM, RS-Transformer, RS-BP, and RS-MLP) employed in rainfall-runoff simulation experiments with a 1-h lead time. The left plot illustrates the distribution characteristics of training time consumption, while the right plot summarizes the maximum, minimum, and average training times. In general, the models can be ranked in descending order of training time as follows: RS-LSTM-Transformer > RS-Transformer > RS-LSTM > RS-BP > RS-MLP, primarily influenced by the complexity of the model structure. Despite a slight increase in training time for RS-LSTM-Transformer compared to RS-Transformer, the former exhibits a more concentrated distribution of training time results.

This phenomenon arises from incorporating the LSTM layer into the input part of the Transformer in the RS-LSTM-Transformer model. The LSTM layer initially processes the input data and selects important information to pass on to the subsequent Transformer layer. As a result, the amount of information and complexity that the Transformer needs to handle is reduced, potentially improving the training efficiency of the model and facilitating convergence, leading to more stable training results.

### Universality of the RS-LSTM-transformer model

To assess the versatility of the RS-LSTM-Transformer model, we conducted a study forecasting flood events in the Guxian watershed in Luohe river using the RS-LSTM-Transformer and compared its performance with the LSTM-Transformer and Transformer models. The Guxian watershed in the Luo River, along with the Jingle watershed, features different climates, land uses, and hydrological characteristics. Supplementary Figs. 2, 3, and Table 3 provide a comparison of flood forecasting results for the RS-LSTM-Transformer model in the Guxian watershed of the Luo River, demonstrating superior performance compared to the Jingle watershed of the Yellow River. In the Guxian watershed, at 1 h of lead time, the calibration and validation periods for the RS-LSTM-Transformer showed NSE, RMSE, MAE, and Bias values of 0.991, 2.489 m^3^/s, 2.102 m^3^/s, 0.445% and 0.989, 4.128 m^3^/s, 4.068 m^3^/s, 0.487% respectively, indicating highly accurate predictions.

## Conclusions

We proposed a hybrid model, the RS-LSTM-Transformer, to improve rainfall-runoff process simulation. This model, which incorporates the framework of the Transformer model, includes an LSTM layer and tunes its parameters using the Randomized Search Optimization technique. The effectiveness of the model is validated through 98 measured flood instances. For lead times ranging from 1 to 6 h, the RS-LSTM-Transformer outperformed the RS-LSTM, RS-Transformer, RS-BP, and RS-MLP models, consistently exhibiting superior performance. It achieved an NSE exceeding 0.875 during the validation phase, with RMSE, MAE, and Bias maintained below 36 m^3^/s, 27 m^3^/s, and 10%, respectively. As the lead time increases, the predictive accuracy of various models diminishes. Yet, the RS-LSTM-Transformer model exhibits a modest downward trend, highlighting its robustness and stability.

In general, the RS-LSTM-Transformer model achieves promising flood forecasting results in the Jingle watershed of the dynamic Yellow River, which is characterized by rapid changes in underlying surface conditions. This highlights its potential in predicting extreme flood events. The RS-LSTM-Transformer model significantly enhances the accuracy and stability of flood forecasting through its hybrid architecture. The application of random search techniques has effectively resolved the challenges of parameter selection in machine learning models. However, the performance of the model depends on high-quality data, and it faces challenges related to computational resource demands and limited interpretability.

In this study, several pressing issues remain to be addressed, particularly how to further reduce forecast uncertainty and enhance model accuracy and stability over longer forecast horizons. One potential direction for future research could involve integrating physics-based models with deep learning techniques. By incorporating environmental factors such as soil moisture, evaporation, and temperature, a more robust forecasting system could be developed. Additionally, we plan to test the model's transferability across different hydro-meteorological conditions to establish algorithmic robustness.

## Supplementary Information

Supplementary Information.

## Data Availability

The datasets analyzed during the current study are not publicly available but are available from the corresponding author on reasonable request.
