# Supplementary material for: Application of a hybrid algorithm of LSTM and Transformer based on random search optimization for improving rainfall-runoff simulation
Source: Sci Rep. 2024 May 16;14:11184. doi: 10.1038/s41598-024-62127-7 (PMC11636842; doi:10.1038/s41598-024-62127-7)
Supplement: Supplementary file 1 — Supplementary Information. [file 41598_2024_62127_MOESM1_ESM.pdf]

Application of a hybrid algorithm of LSTM and Transformer based on random search optimization for improving rainfall-runoff simulation

Wenzhong Li [a], Chengshuai Liu [a*], Caihong Hu [a*], Chaojie Niu[a], Runxi Li[a], Ming Li[a], Yingying Xu[a], Lu Tian[a]

1 Comparison of Model Prediction Performance Under Different Parameter Optimization Methods

As shown in Supplementary Table 1, during the calibration and validation periods, the performance of five models in runoff forecasting (with lead times of 1 hour, 3 hours, and 6 hours) was assessed. The data shown reflect the forecasting effectiveness of the models under initial parameter settings, before any tuning was performed. The comparisons show that the LSTM-Transformer model demonstrated the best forecasting performance at T=1h. At this time point, during the calibration and validation periods, the *NSE*, *RMSE*, *MAE*, and *Bias*% were 0.855, 21.890 m^3/s, 15.978 m^3/s, 1.459% and 0.820, 20.025 m^3/s, 17.001 m^3/s, 1.290%, respectively, indicating that the predictions were very close to actual conditions. At T=6h, the forecasting accuracy of the LSTM-Transformer model declined, with the *NSE*, *RMSE*, *MAE*, and *Bias*% for the calibration and validation periods recorded as 0.821, 396.765 m^3/s, 29.041 m^3/s, 13.158% and 0.800, 38.778 m^3/s, 31.089 m^3/s, 14.115%, respectively.

Supplementary Table 1. compares the performance of five models in flood forecasting (with lead times of 1 hour, 3 hours, and 6 hours) during calibration and validation

periods (under initial parameter settings)

Lead Time(h)	Model	Calibration				Validation			
		NSE	*RMSE*/($m^3 \cdot s^{-1}$)	*MAE*/($m^3 \cdot s^{-1}$)	*Bias*%	*NSE*	*RMSE*/($m^3 \cdot s^{-1}$)	*MAE*/($m^3 \cdot s^{-1}$)	*Bias*%
1h	MLP	0.810	32.550	24.158	5.977	0.789	31.489	29.114	6.978
	BP	0.802	33.987	24.197	4.087	0.705	32.135	26.177	6.007
	Transformer	0.818	24.015	16.008	2.999	0.801	23.789	19.471	2.917
	LSTM	0.801	23.889	19.956	2.984	0.805	22.123	19.067	2.802
	LSTM - Transformer	0.855	21.890	15.978	1.459	0.820	20.025	17.001	1.290
3h	MLP	0.785	41.258	33.897	17.138	0.745	40.283	39.813	16.117
	BP	0.798	40.588	32.014	16.147	0.765	39.512	36.087	15.911
	Transformer	0.851	31.015	26.172	13.173	0.812	28.035	28.127	13.979
	LSTM	0.828	28.454	18.101	18.197	0.881	27.428	18.179	21.712
	LSTM - Transformer	0.840	26.789	11.415	10.137	0.815	25.138	15.401	10.107
6h	MLP	0.699	79.985	69.159	29.123	0.675	77.123	67.138	29.279
	BP	0.700	75.789	68.325	28.017	0.701	74.124	66.889	27.071
	Transformer	0.802	54.168	49.158	15.187	0.741	52.112	44.138	18.901
	LSTM	0.771	50.468	38.097	15.899	0.751	47.497	38.014	18.801
	LSTM - Transformer	0.821	39.765	29.041	13.158	0.800	38.778	31.089	14.115

Supplementary Table 2 demonstrates the performance changes following random adjustments of the model's hyperparameters through a trial-and-error approach. A significant enhancement in overall performance is evident, particularly in metrics such as *NSE*, *RMSE*, *MAE*, and *Bias*. Despite some parameter combinations exhibiting anomalies, it is apparent that random parameter tuning does not fully control the cumulative impact across various performance indicators. For instance, the *NSE* and *RMSE* values for the TAE-LSTM model at forecast horizons of one and three hours were 0.858 and 20.541 m^3/s, and 0.872 and 19.014 m^3/s, respectively, with the model underperforming at a one-hour lead time compared to three hours. Nonetheless, better

outcomes were observed for *MAE* and *Bias* within the same forecast period, recorded at 13.789 m^3/s and 1.554%, and 18.178 m^3/s and 9.159% respectively. Furthermore, the TAE -LSTM-Transformer model exhibited an exceptional performance in *NSE* at a three-hour lead time, with a value of 0.925.

The trial-and-error method involves randomly adjusting parameters to monitor changes in model performance, but this approach struggles to consider multiple performance metrics simultaneously, such as *NSE*, *RMSE*, *MAE*, and *Bias*. This means that even if a particular parameter configuration excels in one metric, it may underperform in others, thereby hindering comprehensive optimisation of model performance. The trial-and-error method often overlooks the complex interactions between parameters and is random in nature, making it difficult to capture performance improvements resulting from specific parameter combinations. In contrast, the RS hyperparameter optimisation algorithm used in the main text not only takes into account a variety of performance metrics but also ensures that *NSE*, *RMSE*, *MAE*, and *Bias* all reach their optimal levels simultaneously, offering a more holistic evaluation of performance.

Supplementary Table 2. Performance comparison of the five models in runoff prediction (lead time = 1h, 3h, and 6h) during calibration and validation periods (Random hyperparameter tuning)

Lead Time(h)	Model	Calibration				Validation			
		NSE	*RMSE*/($m^3 \cdot s^{-1}$)	*MAE*/($m^3 \cdot s^{-1}$)	*Bias*%	*NSE*	*RMSE*/($m^3 \cdot s^{-1}$)	*MAE*/($m^3 \cdot s^{-1}$)	*Bias*%

1h	TAE-MLP	0.852	29.528	21.799	2.977	0.830	32.489	29.745	5.078
	TAE -BP	0.84 5	30.045	23.458	2.087	0.838	31.489	24.489	4.700
	TAE -Transformer	0.861	20.345	15.856	1.987	0.860	21.189	16.489	2.587
	TAE -LSTM	0.858	20.541	13.789	1.554	0.854	20.489	18.789	1.999
	TAE -LSTM -Transformer	0.890	20.052	9.358	0.988	0.908	19.789	15.544	1.258
3h	TAE -MLP	0.810	37.456	31.312	14.025	0.810	39.138	37.489	16.189
	TAE -BP	0.812	38.148	31.048	13.587	0.812	39.178	34.048	14.788
	TAE -Transformer	0.875	29.017	24.138	10.185	0.843	30.178	26.101	13.895
	TAE -LSTM	0.872	19.014	18.178	9.159	0.871	22.078	17.117	12.018
	TAE -LSTM -Transformer	0.938	14.758	13.489	6.785	0.925	13.025	14.489	8.179
6h	TAE -MLP	0.745	79.005	67.787	27.489	0.744	79.994	76.789	29.198
	TAE -BP	0.750	73.138	62.998	24.051	0.748	75.148	62.748	25.017
	TAE -Transformer	0.815	53.179	47.749	14.178	0.814	54.178	47.489	16.015
	TAE -LSTM	0.891	45.148	36.001	13.489	0.830	45.487	36.018	15.458
	TAE -LSTM -Transformer	0.845	34.507	28.024	10.558	0.834	35.198	29.478	12.144

2 Study on the Universality of the RS-LSTM-Transformer Model

2.1 Case study and Data Selection

To assess the versatility of the RS-LSTM-Transformer model, we conducted a study forecasting flood events in the Guxian watershed in Luohe river using the RS-LSTM-Transformer and compared its performance with the LSTM-Transformer and Transformer models.The Guxian watershed in the Luo River, along with the Jingle watershed, features different climates, land uses, and hydrological characteristics.

The study case focuses on the watershed controlled by the Guxian Reservoir, spanning across Shaanxi and Henan provinces, situated between longitudes 109°7' and 111°4' and latitudes 33°7' and 34°4'. The watershed covers an area of 4,759 km². This

region experiences a warm temperate mountain monsoon climate with annual rainfall ranging from 800 to 900 mm, subject to significant interannual variability, which frequently leads to flooding. This paper utilizes data from 24 rain and hydrological stations upstream of the Guxian Reservoir to simulate and forecast flood events from 1990 to 2016, with an hourly time step. The dataset comprises 49 flood events, where 39 events from 1990 to 2011 serve as the training set and the remaining 10 events from 2011 to 2016 as the validation set. As shown in Supplementary Fig. 1, the hydrographic map of the Luo River Guxian watershed location.

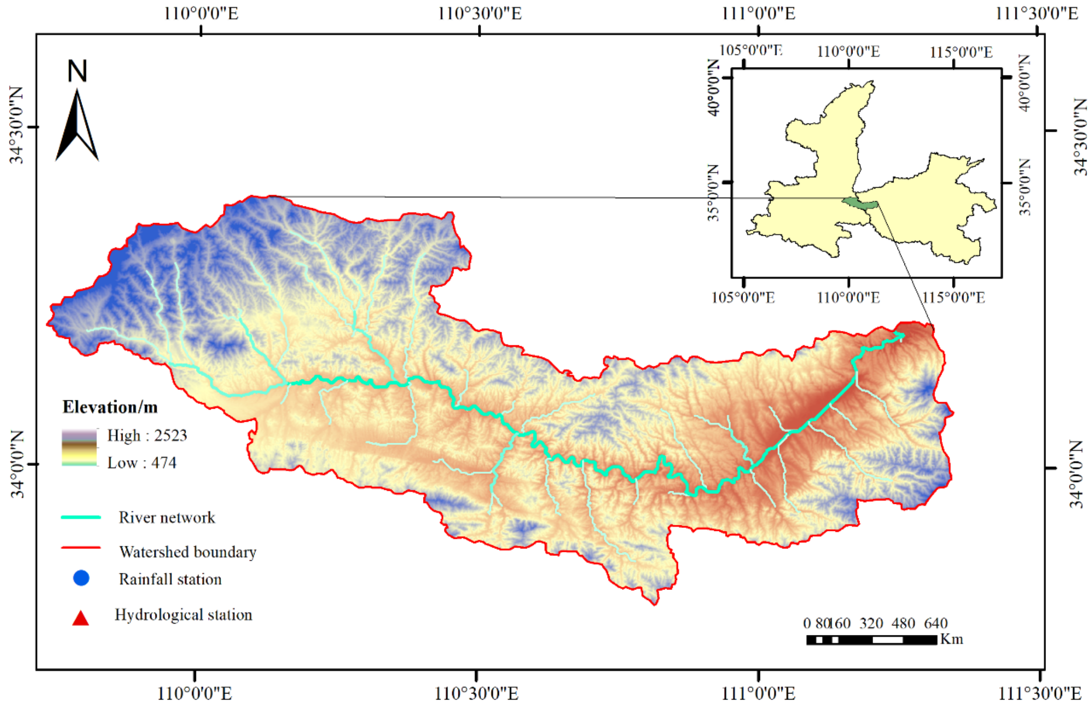

Supplementary Fig.1. The hydrographic map of the Luo River Guxian watershed location

2.2 Results and discussion

2.2.1 Overall Forecasting Performance Evaluation

As shown in Supplementary Fig. 2, it displays the rainfall-runoff process line graphs for the RS-LSTM-Transformer, LSTM-Transformer, and Transformer models over lead times of 1, 3, and 6 hours. The forecasted flow process lines closely align with the observed flow process lines.

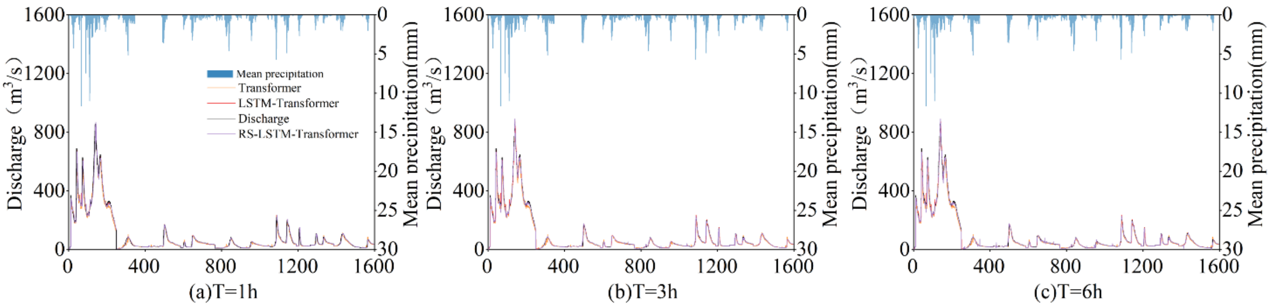

Supplementary Fig. 2. Comparison of overall flood forecasting results for three models with lead times of 1, 3, and 6 hours during the validation period.

As shown in Supplementary Table 3, the statistical evaluation metrics for the forecasting performance of three models at lead times of 1, 3, and 6 hours are presented. The results show that at T=1h, the RS-LSTM-Transformer model exhibits the best forecasting performance, with calibration and validation phase *NSE*, *RMSE*, *MAE*, and *Bias* values at 0.991, 2.489 m^3/s, 2.102 m^3/s, 0.445% and 0.989, 4.128 m^3/s, 4.068 m^3/s, 0.487%, respectively, indicating highly accurate predictions close to reality. At T=6h, the accuracy of the RS-LSTM-Transformer model declines, with *NSE*, *RMSE*, *MAE*, and *Bias* values during calibration and validation phases of 0.945, 19.035 m^3/s, 18.147

m³/s, 5.015% and 0.938, 20.189 m³/s, 21.497 m³/s, 6.489%, respectively. Compared to the LSTM-Transformer and Transformer models, the RS-LSTM-Transformer model achieves superior forecasting precision across all metrics at the same lead times, demonstrating its enhanced nonlinear modeling capability in handling rainfall and runoff dynamics.

Supplementary Table 3. The statistical evaluation metrics for the forecasting performance of three models at lead times of 1, 3, and 6 hours

Lead Time(h)	Model	Calibration				Validation			
		NSE	*RMSE*/($m^3 \cdot s^{-1}$)	*MAE*/($m^3 \cdot s^{-1}$)	*Bias*%	*NSE*	*RMSE*/($m^3 \cdot s^{-1}$)	*MAE*/($m^3 \cdot s^{-1}$)	*Bias*%
1h	Transformer	0.861	20.345	15.856	1.987	0.860	21.189	16.489	2.587
	LSTM - Transformer	0.890	20.052	9.358	0.988	0.908	19.789	15.544	1.258
	RS-LSTM - Transformer	0.991	2.489	2.102	0.445	0.989	4.128	4.068	0.487
3h	Transformer	0.875	29.017	24.138	10.185	0.843	30.178	26.101	13.895
	LSTM - Transformer	0.938	14.758	13.489	6.785	0.925	13.025	14.489	8.179
	RS-LSTM - Transformer	0.978	10.073	7.007	1.123	0.961	13.145	9.489	1.188
6h	Transformer	0.815	53.179	47.749	14.178	0.814	54.178	47.489	16.015
	LSTM - Transformer	0.891	45.148	36.001	13.489	0.830	45.487	36.018	15.458
	RS-LSTM - Transformer	0.945	19.035	18.147	5.015	0.938	20.189	21.497	6.489

2..2.2 Comparison of Single-Event Flood Forecasting Effectiveness

To further understand the model's predictive accuracy for flood events, an analysis was conducted on the rainfall-runoff process line comparisons during two typical flood

events at lead times of 1, 3, and 6 hours. The events analyzed were the flood of September 8, 2015 (Flood 1) and the flood of July 11, 2016 (Flood 2). Supplementary Fig. 3 illustrates that the Transformer model tends to produce more fluctuations before the flood peak in smaller flow flood simulations. The LSTM-Transformer model shows minor error fluctuations at the onset of the flood simulation, possibly due to the memory units retaining characteristics from previous floods. In contrast, the RS-LSTM-Transformer model's forecasts are closest to the observed flow rates, effectively predicting flood peaks and exhibiting good performance during the recession stages of the flood. Thus, the RS-LSTM-Transformer model demonstrates the most stable overall forecasting performance and superior flood prediction capabilities.

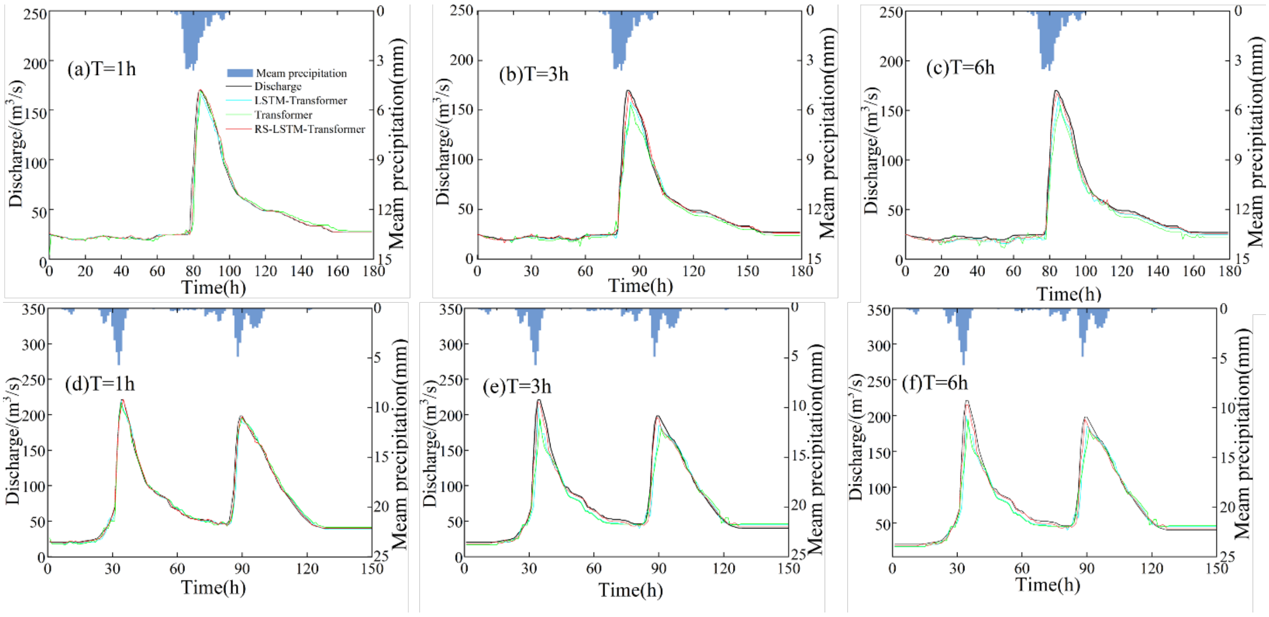

Supplementary Fig. 3. Comparison of Observed Flow Rates and Forecast Results from Transformer, LSTM-Transformer, and RS-LSTM-Transformer Models（Flood event1、2）
